# Frequency and Neural Correlates of Pauses in Patients with Formal Thought Disorder

**DOI:** 10.3389/fpsyt.2013.00127

**Published:** 2013-10-10

**Authors:** Kazunori Matsumoto, Tilo T. J. Kircher, Paul R. A. Stokes, Michael J. Brammer, Peter F. Liddle, Philip K. McGuire

**Affiliations:** ^1^Department of Psychosis Studies, Institute of Psychiatry, King’s College London, London, UK; ^2^Department of Preventive Psychiatry, Tohoku University Graduate School of Medicine, Sendai, Japan; ^3^Department of Psychiatry and Psychotherapy, Philipps-University Marburg, Marburg, Germany; ^4^Centre for Affective Disorders, Department of Psychological Medicine, Institute of Psychiatry, King’s College London, London, UK; ^5^Department of Neuroimaging, Institute of Psychiatry, King’s College London, London, UK; ^6^Developmental Psychiatry, University of Nottingham, Nottingham, UK

**Keywords:** schizophrenia, formal thought disorder, pause, language, fMRI, speech planning, speech monitoring

## Abstract

**Background:** Pauses during speech may reflect the planning and monitoring of discourse, two processes putatively impaired in patients with schizophrenia, particularly those with formal thought disorder (FTD). We used functional MRI to examine the neural correlates of between-clause and of filled pauses, which are respectively associated with speech planning and speech monitoring.

**Methods:** BOLD contrast was measured while six schizophrenia patients with FTD and six healthy subjects spoke about Rorshach inkblots. In an event-related design, we examined activity associated with pauses that occurred between clauses and with pauses that were filled.

**Results:** There was no significant group difference in the frequency of between-clause pauses but patients with FTD made strikingly fewer filled pauses than controls. Between-clause pauses were associated with activation in the anterior part of the left superior temporal gyrus (STG) and the left insula in controls and the engagement of these regions was significantly attenuated in patients.

**Conclusion:** The anterior part of the left STG and the left insula are normally involved in both the planning and monitoring of discourse. The attenuated engagement of these regions with between-clause pauses and the striking infrequency of filled pauses in the patients are consistent with cognitive models implicating defective speech planning and speech monitoring in schizophrenia, especially in relation to FTD.

## Introduction

Formal thought disorder (FTD) is manifest clinically as incoherent speech and is a central feature of schizophrenia. Thought disordered speech appears disorganized and lacks a clear theme or goal, suggesting that an impairment in speech planning might be a key underlying factor (1, 2). Coherent discourse also entails the continuous monitoring and editing of the verbal output, such that the intended and articulated speech correspond (3). The articulation of unusual or inappropriate words and the introduction of incongruous topics in thought disordered speech are thought to reflect a deficit in verbal self-monitoring in schizophrenia (4, 5).

Although normal discourse involves speech that is described as continuous, as much as 50% of the total speaking time can be taken up by pauses which typically last 250–3000 ms (6). Psycholinguistic research indicates that pauses are heterogeneous, with different types associated with specific components of linguistic processing (6–8). Pauses that occur between clauses are particularly associated with planning the content and grammatical structure of the succeeding utterance, while pauses occurring within clauses are more linked to lexical retrieval or word selection (3, 6, 9, 10). Pauses can alternatively be subcategorized according to whether they are filled (and associated with the articulation of “um,” “ah,” and other “fillers”) or unfilled (silent). Filled pauses often reflect the detection of potential errors before they are articulated and may thus indicate the process of verbal self-monitoring (7, 11, 12).

The aim of the present study was to use between-clause and filled pauses as markers of speech planning and monitoring, respectively, in order to study the neural correlates of these processes with functional neuroimaging. We were particularly interested in verbal planning and monitoring because impairments in these processes have been implicated in schizophrenia, especially in relation to FTD. We therefore selected patients who not only had marked evidence of FTD, but who were displaying these symptoms at the time of scanning. On the basis of data from studies of verbal planning and monitoring in other contexts (13–22), we predicted that in controls both types of pause would be associated with activation in the left inferior frontal and left superior temporal cortex. We then tested the hypothesis that between-clause pauses and filled pauses in schizophrenic patients with FTD would be associated with attenuated activation in these regions. This was based on the putative impairments in speech planning and verbal self-monitoring in patients with FTD and previous evidence linking FTD to functional and structural abnormalities in these regions in schizophrenia (23–29).

## Materials and Methods

### Subjects

#### Patients with FTD

Initially, 46 patients with signs of positive thought disorder were identified by clinical teams in the South London and Maudsley NHS Trust, London, UK and referred to the investigators. Of these, 13 were too unwell to tolerate scanning, 10 had only mild FTD, 4 were not native English speakers, 5 were left handed, and 8 declined to participate. The remaining six patients participated in scanning. These subjects met DSM-IV criteria for schizophrenia, were currently exhibiting prominent signs of “positive” FTD and were right-handed (30) native English speakers. The severity of their symptoms on the day of scanning was formally assessed using the Schedule for Affective Disorder and Schizophrenia-Lifetime version (31), the Scale for the assessment of Positive Symptoms (SAPS) (32), and the Scale for the Assessment of Negative Symptoms (SANS) (33). The subjects were included into the study if they had scores of three or more in the SAPS global rating of positive FTD. The mean score of global rating of positive FTD was 3.67, SD = 0.52. The mean score of other positive FTD items were derailment 3.00, SD = 1.26, tangentiality 3.33, SD = 0.82, incoherence 3.17, SD = 0.41, illogicality 2.50, SD = 1.52, circumstantiality, 3.33, SD = 1.21, pressure of speech 1.17, SD = 1.17, distractible speech 2.83, SD = 0.41, and clanging 3.83, SD = 0.75. They had relatively low levels of hallucinations (mean score of global rating of hallucinations 0.33, SD = 0.52), delusions (mean score of global rating of delusions 0.83, SD = 0.75), and negative symptoms (mean global summary score of SANS score 3.33, SD = 2.34, those of SAPS score 6.33, SD = 0.82). All patients were taking stable doses of typical antipsychotic medication (one on chlorpromazine, one on haloperidol, one on sulpiride, one on clozapine, one on chlorpromazine and droperidol, and one on haloperidol and flupentixol). Mean dose in chlorpromazine equivalent was 1042 mg/day (SD = 738).

#### Controls

Six healthy male volunteers who were native English speakers and right handed were recruited from the same geographical area as the patients. They were matched to the patient group for age and socio-demographic variables (Table 1).

**Table 1:** **Sociodemographic and clinical characteristics of patients and controls**.

	Patients with FTD	Control subjects	*p*
Age, mean (SD) (years)	34.3 (11.5)	34.0 (7.9)	0.95
National Adult Reading Test IQ, mean (SD)	101.2 (10.7)	107.6 (9.6)	0.28
Digit span, mean (SD), digits	6.0 (1.9)	7.7 (1.9)	0.13
Continuous Performance Test, mean (SD), errors	3.3 (1.6)	1.9 (0.9)	0.15
Years of full-time education, mean (SD)	11.7 (1.7)	13.3 (2.7)	0.24
Educational achievement, median (range)	3 (1–4)	3 (1–5)	0.8
Best ever occupation, median (range)	3 (3–4)	3 (1–3)	0.7

### Neuropsychological assessment

Verbal IQ, immediate memory recall, and attention were assessed on the day of scanning in all subjects, using the National Adult Reading Test (34), Digit span (35), and the Continuous Performance Test (36), respectively.

The project was approved by the Research Ethical Committee of the Institute of Psychiatry. After complete description of the study to the subjects, written informed consent was obtained.

### Procedure

Subjects were given a standard set of verbal instructions about the experiments and performed three practice trials of the task (using different stimuli from those presented during scanning) 1–5 days before and then immediately before scanning. During scanning, seven Rorschach inkblot plates were presented on a screen viewed via a mirror. These have previously been found to evoke FTD in patients with schizophrenia (26, 37). Subjects were asked to speak about whatever came to mind on viewing the inkblot and to maintain their gaze on the screen. Subjects spoke freely, and no prompting was given if they paused or stopped. Each plate was presented for 3 min (one run), with breaks of approximately 1 min between each presentation (total discourse time: 21 min per subject). Subjects’ speech was recorded on a computer using a non-metallic microphone. Subjects wore customized headphones that reduced the noise of image acquisition but allowed them to hear themselves speak.

### Analysis of verbal responses

Commercially available software (Cool Edit 96; Syntrillium Software Corp., Phoenix, AZ, USA) used to filter out acoustic scanner noise so that the recorded speech was more audible. Subjects’ speech was transcribed from the recordings verbatim by a secretary who was blind to the nature of the study. The transcripts were then examined in conjunction with the speech recordings by an investigator (PS) who was blind to nature and purpose of the study, the types of subjects involved and group status. He was instructed to identify and measure all pauses within each speech sample. Pauses during spontaneous speech do not usually last for more than 3 s. Once the duration of silence is very long, it is questionable whether it really represents a pause in speech (6). Very brief pauses (<250 ms) were considered to be the gaps in phonation associated with adjustment of the position of articulation (6). We therefore operationally defined pauses as the absence of any verbal output for 250–3000 ms. They were subdivided according to two different classification schemes. The first categorized pauses as occurring either between or within clauses. The investigator identified clauses, subordinate clauses, and sentence boundaries, based on the transcriber’s punctuation and carefully listening to the corresponding recording. The second scheme classified pauses as either filled (with non-word sounds, like “um” or “ah”) or unfilled (entirely silent). Filled pauses comprised pauses in which the sounds occurred in the middle of the pause, or merged with either the end of a preceding word or with the beginning of the next word.

Cool Edit software was used to mark the point of onset and measure the duration of each pause in each time series. All pauses were included in the analysis of the speech samples but extremely short (<250 ms) or long (>3000 ms) pauses were excluded from the neuroimaging analyses. To achieve sufficient power to detect activation associated with between-clause and filled pauses, we analyzed all pauses between 250 and 3000 ms duration. This approach differed from that employed in a previous study (16) in which only 85 longest pauses between 550 and 3000 ms duration in each subject were selected. In the present study, the mean number of pauses analyzed per group was 389.5 (SD = 60.7) in controls and 251.3 (SD = 110.9) in patients.

The subjects’ speech was separately evaluated with respect to the severity of FTD using the Thought and Language Index (TLI) (38) by another investigator (Peter F. Liddle) who was trained in the use of the TLI but was blind to subject identity and group status.

### Image acquisition

Gradient-echo echoplanar MR images were acquired using 1.5 T GE Sigma System (General Electric, Milwaukee, WI, USA) fitted with Advanced NMR hardware and software. In each of 14 non-contiguous planes parallel to the AC-PC plane, 60 T2*-weighted MR images depicting BOLD contrast (39) were acquired with TD = 40 ms, TR = 3000 ms, θ = 90X, in-plane resolution = 3.1 mm, slice thickness = 7 mm, slice skip = 0.7 mm. Head movement was limited by foam padding within the head coil and a restraining band across the forehead. For anatomical coregistration, a 43-slice inversion recovery echoplanar image of the whole brain was acquired (echo time, 73 ms; inversion time, 180 ms; repetition time, 16,000 ms; in-plane resolution, 1.5 mm; slice thickness, 3 mm; and slice skip 0.3 mm).

### Image analysis

Each series of images acquired during the seven runs in each subject was analyzed separately. The point of onset of each pause type was noted in each time series. To take into account the potential effect of variation in pause duration on the associated activation, each pause was characterized in terms of the number of 250 ms subunits it comprised. Each subunit was then treated as an event of interest. The BOLD response associated with pauses of duration 250–3000 ms (the events of interest) was contrasted with a baseline condition comprising the response during continuous speech plus that during pauses that were either shorter than 250 ms or longer than 3000 ms. The same baseline was also used in the contrasts involving subtypes of Pauses. Thus (between-clause pauses vs. baseline) was compared with (within-clause pauses vs. baseline), and (filled pauses vs. baseline) was compared with (unfilled pauses vs. baseline).

Before analysis, the effects of small amounts of subject motion during data acquisition were corrected using a two-stage process involving realignment and regression (40). In computing the correlation between behavioral and imaging data, it was necessary to minimize the possibility of spurious correlations leading to type I errors. Such effects are most likely to occur if the behavioral data show a simple monotonic trend, which could show apparent correlations with drifts in image intensity. To deal with this possibility, the seven-3 min runs of behavioral data obtained from each individual were examined, and the two runs with highest intrarun variance and at least two maxima and two minima were selected for correlational analysis. Two runs were used because all subjects had at least this number of runs showing clearly non-monotonic time and behavior characteristics.

### Individual analysis

The data were realigned (41) and smoothed using a Gaussian filter (FWHM 7.2 mm). Event-related activation was detected in a time-series analysis using Gamma variate functions (peak responses at 4 and 8 s) to model the BOLD response. First, the BOLD response associated with each type of pause was separately convolved with 4 and 8 s Poisson functions to yield two models of the expected hemodynamic response. The weighted sum of these two convolutions that gave the best fit to the time series at each voxel was then computed. This weighted sum effectively allows voxel-wise variability in time to peak hemodynamic response. In order to constrain the possible range of fits to a physiologically plausible BOLD response, the constrained fitting procedure suggested by Friman et al. (42) was adopted. A goodness of fit statistic (the SSQratio) was then computed at each voxel: the ratio of the sum of squares of deviations from the mean intensity value due to the model (fitted time series) divided by the sum of squares due to the residuals (original time series minus model time series).

To sample the distribution of SSQratio under the null hypothesis that observed values of SSQratio were not determined by experimental design, the time series at each voxel was permuted using a wavelet-based resampling method (43, 44). This process was repeated 10 times at each voxel and the data combined over all voxels, resulting in 10 permuted parametric maps of SSQratio at each plane for each subject. The same permutation strategy was applied at each voxel to preserve spatial correlational structure in the data during randomization. Combining the randomized data over all voxels yielded the distribution of SSQratio under the null hypothesis. A test that any given voxel was activated was carried out by obtaining the appropriate critical value of SSQratio from the null distribution.

### Group mapping

The observed and randomized SSQratio maps were transformed into standard space by a rigid body transformation of the fMRI data into a high-resolution inversion recovery image of the same subject followed by an affine transformation onto a Talairach template (40). This generated a generic brain activation map (GBAM) for each experimental condition. The median observed SSQratio over all subjects at each voxel were then tested at each intracerebral voxel against a critical value of the permutation distribution for median SSQratio ascertained from the spatially transformed wavelet-permuted data (40). To increase sensitivity and reduce the risk of type I errors testing was carried out at the cluster level (45). This estimated the probability of occurrence of clusters under the null hypothesis using the distribution of median SSQratios computed from the time series at each voxel (see above). Image-wise expectation of the number of false positive clusters under the null hypothesis was set at <1.

### Group differences

Analysis of variance was carried out on the SSQratio maps by first computing the difference in median SSQratio between groups at each voxel. Inference of the probability of this difference under the null hypothesis was made by reference to the null distribution obtained by repeated random permutation of group membership and recomputation of the difference in median SSQratios between the two groups obtained from the resampling process. Cluster-level maps were then obtained as described above.

## Results

### Sociodemographic and neuropsychological data

There were no significant group differences on socio-demographic variables or neuropsychological performance (Table 1).

### Behavioral data

In one patient their speech during three of the runs was not clearly audible. Another patient failed to speak during one run. Data from these runs were excluded from the analyses.

Pauses (all types summed together) occurred more frequently in controls than patients. This reflected a much lower frequency of filled pauses in the patient group (Table 2). This difference in the frequency of filled pauses between groups remained significant after covarying for the total number of words produced. There were no significant group differences in the frequency of unfilled, between-clause, or within-clause pauses. Although all types of pause tended to be longer in patients than controls, these differences were not statistically significant (Table 2). In controls 30.2% (SD = 14.6) of between-clause pauses were filled while 45.6% (SD = 15.3) of filled pauses occurred between clauses.

**Table 2:** **Number and duration of pauses in patients and controls**.

	Controls	FTD patients	*p
	
	Median (interquartile range)		
**NUMBER OF PAUSES/RUN**			
All	55.9 (9.1)	43.0 (11.2)	0.04
Between-clause	24.3 (7.7)	22.2 (10.4)	0.59
Within-clause	30.8 (10.0)	13.0 (9.6)	0.13
Filled	14.7 (6.2)	0.5 (1.8)	<0.01
Unfilled	40.9 (11.9)	41.6 (11.4)	0.70
**DURATION OF PAUSES (S)**			
All	0.80 (0.21)	1.15 (0.20)	0.09
Between-clause	1.03 (0.29)	1.33 (0.27)	0.18
Within-clause	0.64 (0.12)	0.84 (0.20)	0.24
Filled	1.25 (0.21)	1.65 (0.60)	0.39
Unfilled	0.65 (0.16)	1.10 (0.28)	0.07

The severity of positive FTD (as indexed by the total TLI score per run) during scanning ranged from 0.1 to 34.4 in patients and from 0.1 to 4.1 in controls, with mean scores of 9.7 (SD = 12.5) and 1.4 (SD = 1.5) respectively. There was a trend for an inverse correlation between the severity of FTD and the number of filled pauses per run (Spearman’s correlation; *r* = −0.53, *p* = 0.075).

### Head movement

Head movement was assessed in each subject by examining two runs from the seven in each individual. The maximum amount of head movement in the three dimensions (*x*, *y*, *z*) in the patients was *x*: 0.6 (SD = 0.2) mm, *y*: 0.6 (SD = 0.3), *z*: 1.5 (SD = 1.5) and in the controls was *x*: 0.3 (SD = 0.2), *y*: 0.4 (SD = 0.4), *z*: 0.9 (SD = 0.7).

### Activation during pauses

#### Controls

**Figure 1:**
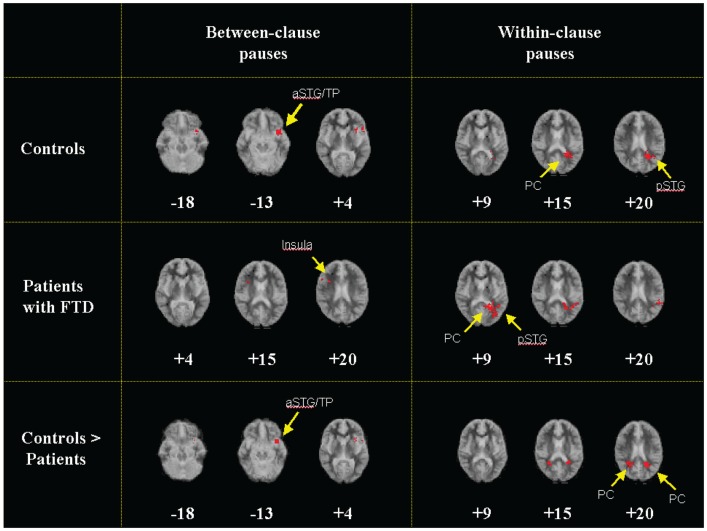
**Generic brain activation maps of activation during pauses between clauses and within clauses in controls (top) and patients (middle) (*p* < 0.002)**. Controls showed greater activation than patients in a region that included the polar part of the left aSTG and the adjacent insula during between-clause pauses and in a region which included the bilateral PC and the pSTG during within-clause pauses (bottom). Talairach *z* coordinates (46) are shown on the bottom of each image. The left side of the map represents the right side of the brain. aSTG, anterior superior temporal gyrus; TP temporal pole; pSTG, posterior superior temporal gyrus; PC, posterior cingulate gyrus.

Between-clause pauses were associated with activation in the anterior part of the superior temporal gyrus (STG), close to the temporal pole (Table 3; Figure 1). Within-clause pauses were associated with activation in a region which included the left posterior cingulate cortex and extended laterally into the posterior part of the left STG. (Table 3; Figure 1).

**Table 3:** **Foci of activation during between- and within-clauses pauses and differences between groups**.

Cerebral region	BA	Side	Talairach coordinates			Cluster size	*p*
			*X*	*Y*	*Z*	
**Generic brain activation maps**
Controls
Between-clause pauses
Superior temporal gyrus/temporal pole	22/38	L	−38	8	−13	86	0.001446
Within-clause pauses
Retrosplenial cingulate gyrus	30	L	−14	−50	20	79	0.000313
FTD patients
Between-clause pauses
Insula/inferior frontal gyrus	44	R	35	8	20	104	0.001725
Within-clause pauses
Retrosplenial cingulate gyrus	29/30	L	−17	−47	9	142	0.001327
**Comparison maps**
Between-clause > within-clause pauses
Controls
Temporal pole	22/38	L	−35	11	−13	80	0.001264
Superior temporal gyrus	22	L	−49	−19	4	102	0.00092
FTD patients
Lingual gyrus	18	L	−12	−53	4	107	0.001695
Cuneus	17	R	20	−72	15	70	0.00113
Between-clause pauses
Controls > FTD patients
Temporal pole	22/38	L	−38	8	−13	63	0.000292
Within-clause pauses
Controls > FTD patients
Retrosplenial cingulate gyrus/posterior superior temporal gyrus	29/39	R	26	−44	20	42	0.001401

When compared directly (Table 3), between-clause pauses were associated with greater activation than within-clause pauses in an area that included the polar part of the left STG and the ventral part of the left insula. They were also associated with greater activation in a separate region in the middle of the left STG. There were no areas that were relatively more activated during within-clause pauses.

**Figure 2:**
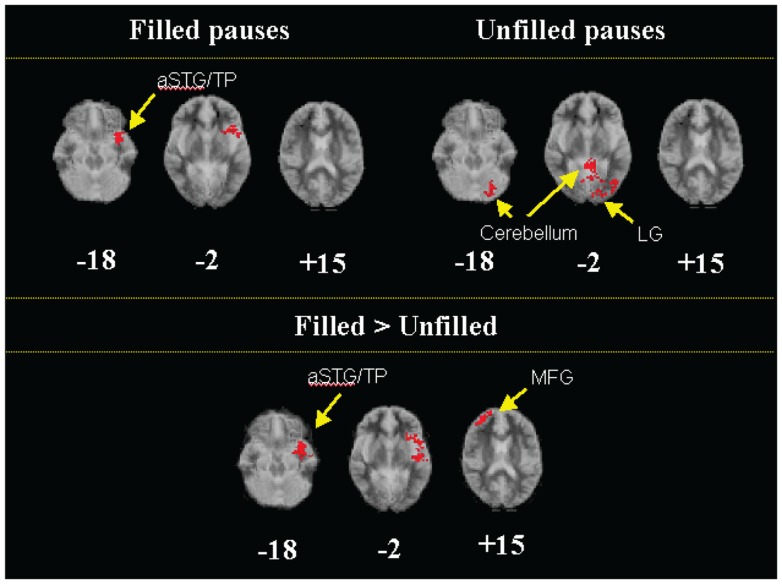
**Generic brain activation maps of activation during filled and unfilled pauses in controls (top) and comparison maps showing greater activation in a region that included the polar part of the left aSTG and the adjacent insula and in the right MFG during filled pauses than unfilled pauses in controls (bottom) (*p* < 0.002)**. aSTG, anterior superior temporal gyrus; TP, temporal pole; LG, lingual gyrus; MFG, middle frontal gyrus.

Filled pauses in controls were associated with activation in the temporal polar part of the left STG, in a region similar to that activated during between-clause pauses (Table 4; Figure 2). In contrast unfilled pauses were associated with activation in a region which included the cerebellum and the left lingual gyrus. When filled and unfilled pauses were compared directly, filled pauses were associated with greater activation in an area that included the polar part of the left STG and extended into the adjacent left inferior frontal gyrus and insula (Table 4; Figure 2). Filled pauses were associated with a further cluster of activation relative to unfilled pauses in the right middle frontal gyrus. There were no areas that were relatively more activated during unfilled pauses.

**Table 4:** **Foci of activation during filled and unfilled pauses and differences between them**.

Cerebral region	BA	Side	Talairach coordinates			Cluster size	*p*
			*X*	*Y*	*Z*	
**Generic brain activation maps**
Filled pauses
Superior temporal gyrus/temporal pole	22/38	L	−35	6	−18	116	0.00016
Unfilled pauses
Cerebellum		L	−3	−47	−2	230	0.000323
**Filled pauses > unfilled pauses**
Insula/temporal pole	22	L	−40	11	−2	233	0.000122
Middle frontal gyrus	46	R	38	39	15	36	0.001955

### Patients with schizophrenia and FTD

In the patients, between-clause pauses were associated with activation in the superior part of the right insula, but there was no engagement of the left STG or left inferior frontal/insular cortex as there was in the controls (Table 3; Figure 1). Within-clause pauses were associated with activation in a region that extended laterally from the left posterior cingulate gyrus to the posterior portion of the left STG, as had been evident in the controls (Table 3; Figure 1). When compared directly (Table 3), between-clause pauses were associated with greater activation than within-clause pauses in the left lingual gyrus and the right cuneus. There were no areas that were relatively more activated during within-clause pauses.

The patients made too few filled pauses to permit an analysis of their neural correlates. Unfilled pauses in the patients were associated with activation in the precentral gyri bilaterally, the right retrosplenial cingulate gyrus, and the brain stem.

### Between-group comparisons

#### Between-clause pauses

Activation in controls was greater than in patients in a region that included the polar part of the left STG and the adjacent anterior insula. There were no areas more activated in patients than controls (Table 3; Figure 1).

#### Within-clause pauses

Controls showed greater activation than patients bilaterally in a region which included the posterior cingulate gyrus and extended laterally into the posterior portion of the STG. No areas were more activated in patients than controls (Table 3; Figure 1).

#### Filled and unfilled pauses

Patients made too few filled pauses to compare the associated pattern of activation between groups.

## Discussion

### Frequency of pauses in patients with formal thought disorder

We selected patients with schizophrenia who had marked FTD as this symptom has been particularly associated with impairments in the planning and the monitoring of speech (1, 2, 4, 5). The most significant finding at the behavioral level was that the patients produced far fewer filled pauses than healthy controls. This difference was specific to filled pauses, with no significant group differences in the frequency of unfilled, between-clause, or within-clause pauses, and accounted for the overall difference in the frequency of pauses in general. A relative lack of filled pauses does not seem to have been observed before, perhaps because previous studies of pauses in schizophrenia have not subcategorized them as filled and unfilled [e.g., Resnick and Oltmanns (47)]. A low frequency of filled pauses is of particular interest in patients with FTD as the production of filled pauses is normally associated with the monitoring of speech for errors or anomalies (7, 12) and cognitive models of schizophrenia propose that a deficit in speech self-monitoring underlies disorganized speech (4). This finding, however, should be considered preliminary because the sample size was small: it requires replication in a larger sample. Future studies could also investigate the neural correlates of pauses in patients with schizophrenia who do not have FTD, to clarify the extent to which the present findings are specific to patients with this particular symptom or are a generic feature of schizophrenia, independent of FTD.

### Neural correlates of between-clause pauses

Consistent with the first hypothesis, between-clause pauses in controls were associated with activation in the left STG, with the focus in the temporal pole. The more controlled comparison with within-clause pauses (as opposed to speech in general) revealed additional activation in the ventral part of the adjacent left insula and in a more posterior region in the middle of the left STG. These differences, relative to another pause type as opposed to the rest of the discourse, indicate that the activation was not secondary to reduced activity in these areas during the non-pause parts of discourse. These findings are consistent with evidence that the left temporal and inferior frontal cortex are normally involved in linguistic processing at the sentence level (13, 17, 18, 21, 22) and in the processing of syntactic information (48, 49). Some authors of the present study have previously reported the results of an fMRI study of pauses from the same group of healthy subjects. However this earlier study used a different method of pause analysis (16). In the present study, in order to compare pauses in FTD patients with those in healthy controls, we modified the methodology: we sampled as many pauses as possible so that we would have sufficient power to detect the activation associated with both between-clause pauses and filled pauses in both groups. Thus, in the present study we were able to analyze the responses associated with many more between-clause pauses (mean 171.7, SD = 49.9) than in the previous study (16) (mean 51.8, SD = 9.1). Without this modification in the methodology, it would not have been possible to analyze between-clause pauses in both groups.

Consistent with our second hypothesis, between-clause pauses in patients with FTD were associated with attenuated engagement of the left anterior temporal cortex relative to controls, as well as reduced activation in the left insula. This was not simply due to a general reduction of activation during pauses in the patient group, as when the patients made within-clause pauses, they showed the same pattern of regional activation as controls. Rather it reflected the engagement of different areas in the patients during between-clause pauses, with activation in the right insula as opposed to the left in controls. Given that between-clause pauses may normally reflect speech planning (6), and that the left temporal and inferior frontal cortex are normally involved in this process (above), the reduced activation in these areas may thus indicate an impairment in discourse planning. Cognitive models of FTD suggest that it is related to an impairment in speech planning (1, 2). Functional neuroimaging studies in schizophrenia have reported that the production of thought disordered speech is associated with reduced activation in the left STG and left inferior frontal/insular cortex (26, 27), while structural imaging data suggest that FTD is associated with reduced left STG (24, 25, 28, 50) and left temporal pole volume (25).

### Neural correlates of within-clause pauses

As reported previously (16), within-clause pauses were associated with posterior part of the left STG. There were also group differences in activation during within-clause pauses, with patients showing a weaker engagement of the posterior parts of the cingulate and superior temporal cortices than controls. These differences had not been predicted *a priori*. As within-clause pauses are normally associated with lexical retrieval (3, 6, 9, 10), this differential activation may reflect the impairment in this process that is evident in schizophrenia (51–53).

### Neural correlates of filled pauses

Filled pauses in controls were associated with engagement of the anterior left STG relative to baseline, and with additional activation in the left insula and the right dorsolateral prefrontal cortex relative to unfilled pauses. Psycholinguistic studies have implicated filled pauses in the monitoring of speech (7, 12). While there have not been neuroimaging studies of discourse monitoring, the monitoring of self-generated verbal material at the single word and the sentence level normally involves the left STG (20, 54).

The similarity between the activation associated with between-clause and with filled pauses in controls, suggests that the left insula and STG may mediate processes common to both discourse planning and monitoring, such as complex semantic and syntactic processing, or the executive control of these processes. It is unlikely that it simply reflects the coincidence of between clause and filled pauses, as most between-clause pauses were unfilled and most filled pauses occurred within rather than between clauses.

The low frequency of filled pauses in the patients precluded analysis of their neural correlates and we could not compare the associated activation with that in the controls.

### Methodology

The size of the patient group in the present study was limited by major logistical constraints. Patients with schizophrenia who exhibit marked FTD are relatively uncommon, and among the subset that display thought disorder the symptoms are usually transient, making it difficult to scan patients who are thought disordered at the time of scanning. Moreover, because thought disorder is associated with behavioral disorganization and distractibility (55), this subgroup is often unable to cooperate with functional imaging paradigms. This was especially true of the paradigm used in the present study, which was particularly demanding, requiring subjects to talk continuously for relatively long periods (3 min) about a series of seven abstract stimuli while lying still inside an MRI camera. Our patients were thus very difficult to recruit, reflected in the fact that an initial sample of 46 patients with thought disorder was needed to yield a group of 6 suitable subjects, and that the recruitment process took over 2 years, despite the patients being drawn from a large psychiatric center. By using a non-parametric method of image analysis we reduced the risk that the group findings would be influenced by statistical outliers.

Thus, while it would be useful to replicate the present findings in a larger patient sample, this may be logistically difficult. However the extent to which our findings are particularly related to FTD could be further investigated by examining patients with schizophrenia who do not have this symptom, and these patients would be easier to recruit and study.

## Conclusion

Filled pauses and between-clause pauses in controls were associated with activation in the anterior part of the left STG and the left insula, which may be a correlate of discourse planning and monitoring. Patients with schizophrenia and FTD showed reduced activation in these regions in association with between-clause pauses and produced far fewer filled pauses than healthy controls. These findings are consistent with cognitive models that propose defective discourse planning and monitoring in FTD in schizophrenia.

## Conflict of Interest Statement

The authors declare that the research was conducted in the absence of any commercial or financial relationships that could be construed as a potential conflict of interest.
